# Research advances in roles of microRNAs in nasal polyp

**DOI:** 10.3389/fgene.2022.1043888

**Published:** 2022-11-25

**Authors:** Niu Zhipu, Huo Zitao, Sha Jichao, Meng Cuida

**Affiliations:** ^1^ Clinical Medicine, China-Japan Union Hospital of Jilin University Norman Bethune Third School of Jilin University, Changchun, China; ^2^ Department of Otorhinolaryngology Head and Neck Surgery, China-Japan Union Hospital of Jilin University Norman Bethune Third School of Jilin University, Changchun, China

**Keywords:** microRNA, chronic rhinosinusitis with nasal polyps, RNA binding protein, inflammatory response, airway remodeling, exosomes

## Abstract

MicroRNAs (miRNAs), a subset of endogenous RNAs highly conservative with short chains, play key regulatory role in the biological relevant events of the cells. Exosomes are extracellular vesicles like the plasma membrane components being able to deliver information molecules such as miRNA between cells and to regulate the fate of the target cells. The progression of chronic rhinosinusitis with nasal polyps (CRSwNP) is closely associated with significant alterations of miRNA levels in both cells and exosomes. RNA-binding proteins (RBPs) have been acknowledged to play important roles in intracellular miRNA transport to exosomes, and specific membrane proteins such as caveolin-1 critically involved in HNRNPA1 -mediated transport of miRNA to exosomes. Aberrant alteration in endogenous miRNA levels significantly contributes to the process of airway remodeling in the nasal tissue and to the occurrence and progression of inflammatory responses in CRSwNP. Exogenous miRNAs delivered *via* exosomes has also been shown to play an important role in activating macrophages or in regulating vascular permeability in the CRSwNP.This paper highlights the mechanism of RBP-mediated delivery of miRNAs to exosomes and the important contribution of endogenous miRNAs to the development of CRSwNP in response to inflammation and airway remodeling. Finally, we discuss the future research directions for regulation of the miRNAs to CRSwNP.Delivery of exogenous miRNAs by exosomes alters the endogenous miRNAs content in nasal mucosal epithelial cells or in associated inflammatory cells in the CRSwNP, and altered endogenous miRNAs affects the inflammatory response and airway remodeling, which then regulates the occurrence and progression of CRSwNP.RBPs and associated membrane proteins such as caveolin-1 may play a crucial role in the entry of exogenous miRNA into exosomes.

## 1 Introduction

**FIGURE 1:**
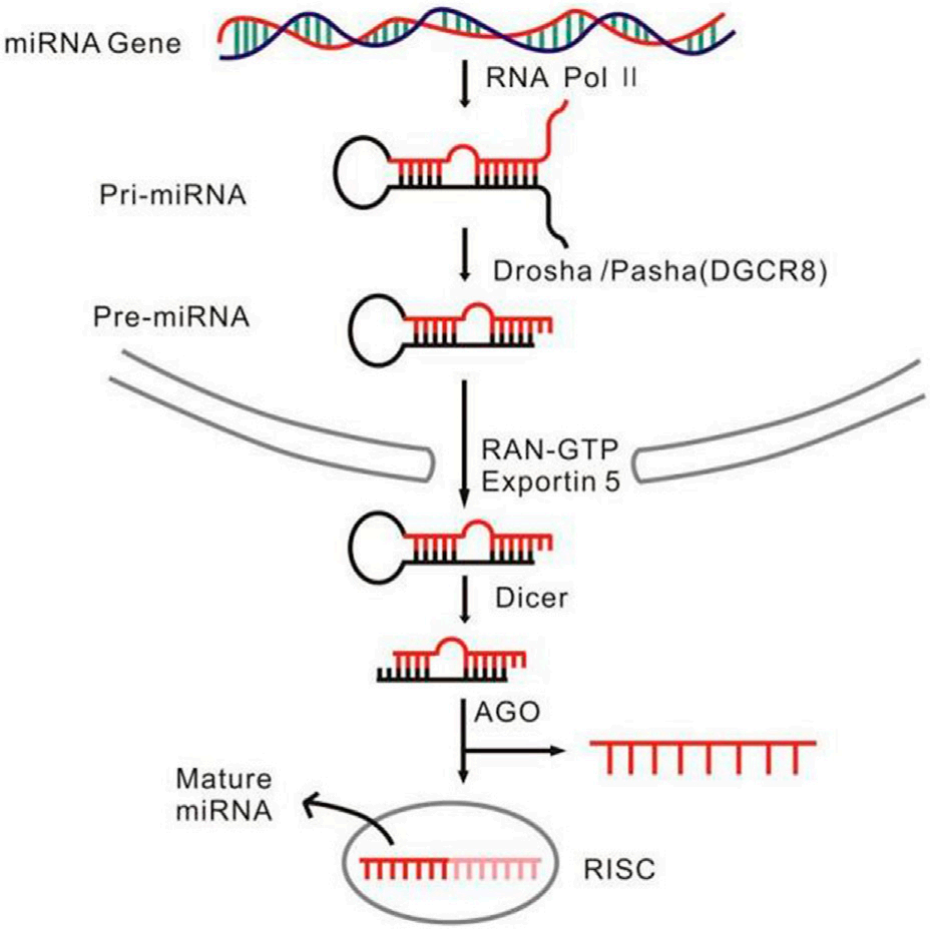
The miRNA formation and function process.

MiRNAs are a set of endogenous single-stranded small molecule RNAs with length of 18–22 nt, non-translatable, and generated *via* two successive steps of processing from primary-miRNAs (pri-miRNAs) to precursors of miRNAs (pre-miRNA) and from pre-miRNAs to mature miRNAs (Lee et al., 2004) (Figure 1). Molecularly, miRNAs regulate gene expression at the transcriptional and post-transcriptional levels, whereas biologically, they are critically involved in almost all the known biological events such as cell proliferation and differentiation, development, reproduction, and aging (Chen and Wang, 2012; Gerasymchuk et al., 2020; Wang et al., 2021). Aberrant alterations of the miRNA abundance and the species often initiate pathogenesis of many diseases, initially making it plausible to speculate that the miRNAs could become the potential biomarkers translatable to clinic therapy for many kind of diseases. Indeed, so far many miRNAs have been acknowledged as the key regulators implicated in various disease such as cancers and neurological disorders, some of which have been developed as therapeutic targets for some stubborn diseases (Sun et al., 2018; Peng et al., 2020).

The abundance of miRNAs in cells is dynamically regulated in accordance with physiological conditions and development stage. Aberrant alteration of miRNAs may significantly contribute to initiation and/or progression of diseases. Several mechanisms account for the dynamic alteration of miRNAs abundance. The common one is up-regulation or downregulation of miRNA genes, while the enhanced or blocked miRNA package into exosomes and transportation to outside cells also become factors.

The occurrence and progression of CRSwNP is associated with alteration of intracellular miRNA levels, although the differential expression of miRNA was also detected in the nasal lavage fluid (NLF)-EV (extracellular vesicles) of CRSwNP patients (Cha et al., 2021). Some miRNAs can regulate the epithelial-mesenchymal transition (EMT) to affect the airway remodeling of CRSwNP (Liu et al., 2021b), of which is miR-155 that could be regulated by glucocorticoids, commonly serving as clinical therapy (Zheng et al., 2012). Both studies demonstrate that miRNA is involved in the development and progression of nasal polyps at both the phenotypic and molecular levels, suggesting the importance of miRNAs in CRSwNP in exploration of the occurrence mechanism of CRSwNP and clinic therapy.

The CRSwNP patients with the prevalence of 0.5%–4.3% developed clinical manifestations such as unconsciousness, dizziness, memory loss and hyposmia, and is often accompanied by asthma comorbidity and postoperative recurrence (Table 1). The CRSwNP mainly showed Th2 cell inflammation, mainly involving the effects of IL-4, IL-5, and IL-13, accompanied by changes in IL-25, IL-33, TSLP and IgE levels, with eosinophil infiltration and nasal polyps phenomenon (Fossiez et al., 1996). Besides, CRSwNP is associated with elevated levels of eosinophils, innate lymphoid cells (ILC2), macrophages and mast cells (Lee et al., 2021). NP is characterized by epithelial cell alterations, epithelial mesenchymal transformation, goblet cell hyperplasia, extracellular matrix degradation, fibrin deposition, and tissue edema.

**TABLE 1:** Introduction of the CRSwNP.

CRSwNP	
prevalence	0.5%–4.3%
clinical manifestation	unconsciousness,dizziness,memory loss and hyposmia
Inflammation type	Th2 cell inflammation
Associated inflammatory substances	IL-4, IL-5 and IL-13, accompanied by changes in IL-25, IL-33, TSLP and IgE levels
Related cells	Nasal epithelial cells, ILC2, eosinophils, mast cell,macrophage,Th1/Th2/Th17/Treg cell
Tissue feature	EMT, airway remodeling, fibrin accumulation,Increased mucus,polyp
Disease feature	Asthma comorbidity and postoperative recurrence

## 2 The function of inflammatory cells in the CRSwNP

One of the important pathological features exhibited by CRSwNP is inflammatory cell infiltration. Infiltrating inflammatory cells include nasal epithelial cells, ILC2, eosinophils, mast cell, macrophage and Th1/Th2/Th17/Treg cell, mostly Th2 cells. The inflammatory cell infiltration is accompanied by changes of cytokines and chemokine expression. Nasal epithelial cells are capable of regulating the development of Type II inflammatory response by releasing TSLP, IL-33 and IL-25. Activated ILC2 leads to eosinophilia mainly by releasing IL-5, whereas release of IL-13 promotes airway hyperresponsiveness, goblet cell hyperplasia, mucus production, and DC activation. Th2 cells releases IL-4, IL-5, and IL-13 participates in the inflammatory response, and eosinophils releases the chemokine CCL23 to recruit M2 macrophages to release chemical mediators, such as histamine, to induce mucosal oedema and local inflammation. Activated macrophages release proinflammatory factors and recruit inflammatory cells to participate in the inflammatory response. The Th1/Th2/Th17/Treg cell balance directed towards Th2 mediates the progression of the type 2 inflammatory response.

### 2.1 Nasal epithelial cells

Nasal epithelium cells in CRSwNP are able to release IL-25, IL-33 and TSLP by which are able to activate type 2 innate lymphoid cells (ILC2) and to increase the production of type 2 cytokines (Fujieda et al., 2019). At the same time, TLSP can activate dendritic cells (DCs) to present antigen and costimulatory signals, and differentiate naive T cells into effector Th2 cells, causing the release of type 2 cytokine to increase (Patel et al., 2019). Type II inflammatory response can damage the epithelial cells of the nasal mucosa, improve the nasal mucosal epithelial permeability, and decrease mucosal barrier function, basement membrane thickening, stromal fibrosis, epithelium to mesenchymal transformation (EMT), airway remodeling and other phenomena.

### 2.2 ILC2

Epithelial-derived cytokines as well as other biological mediators such as lipid mediators enable to activate ILC2. The activated ILC2 can release IL-4, IL-5, IL-8, IL-9, IL-13, GM-CSF, and Th2 cells to enhance type 2 immune responses. Releases of IL-5 and IL-13, but only the IL-5 by the activated ILC2 could raise eosinophil levels in contrast to IL-13 that leads to airway hyper-response (AH), increasing the number of goblet cells and enhancing mucus secretion. Furthermore, IL-4, IL-5 and IL-9 released by the activated ILC2 could induce B cell proliferation and IgE production. By contrast, IL-8 and GM-CSF released by ILC2 activate neutrophils and macrophages (Poposki et al., 2017; van der Ploeg et al., 2020). IL-9 released by IL2 could maintain ILC2 survival in polyp tissue and increase the levels of mast cells. ICL2 releases cytokines, such as IL-2, IL-4, and IL-13, and induces Th2 cell proliferation and promote the occurrence of dimorphic inflammatory response (Li et al., 2021).

### 2.3 Th2 cells, together with eosinophils

DCs stimulate the differentiation of naive T cells into effector Th2 cells, increasing the secretion of type-2 cytokines such as IL-4, IL-5, and IL-13 (Fossiez et al., 1996). While IL-4 promotes IgE production (Punnonen et al., 1997), IL-5 is able to induce the production of eosinophilic extracellular traps (Hamilos et al., 1995). The chemokine eosinophils (Cho-C motif) ligand (CCL) 23 recruits macrophages and differentiate into M2 macrophages. CCL23 released by eosinophils recruits macrophages and differentiates into M2 macrophages (Poposki et al., 2011).

### 2.4 Mast cell

Activated mast cells release chemical mediators, such as histamine, to induce mucosal oedema and local inflammation. Upon stimulation with TSLP, IL-33, and IL-1, mast cells secrete IL-3 and IL-13 (Saluja et al., 2016). Both IgE and mast cells numbers increase in eosinophilic CRSwNP, and IgE is involved in regulating the regulation and activation of mast cell degranulation. Allergens were found to be able to bind to specific IgE on CRSwNP mast cells, activate mast cells and release a variety of pro-inflammatory mediators and cytokines such as prostaglandin D2, prompting the development of an inflammatory response (Cao et al., 2014).

### 2.5 Macrophage

Activated macrophages not only release the proinflammatory cytokines TNF and IL-1, but also could differentiate into M2 macrophages thereby releasing paclitaxel and chemokine CCL18 and recruiting eosinophils, Th2 cells and myeloid dendritic cells involved in inflammatory cell infiltration and inflammatory response of CRSwNP (Peterson et al., 2012).

### 2.6 Th1/Th2/Th17/treg cell

The traditional view is that Th0 cells from CRSwNP patients tend to differentiate into Th2 cells stimulated by inflammatory factors, disrupting the original Th1/Th2 cell balance and mediating the occurrence of type 2 inflammatory response (Omori and Ziegler, 2007). Recent studies have found that the Th17/Treg cell balance has an important role in the occurrence and progression of the inflammatory response. CD4 + T cells differentiate into Th17 cells in the presence of inflammatory factors, and the released IL-17 is able to trigger an inflammatory response (Fossiez et al., 1996), both of which can differentiate into Treg cells and play an immunosuppressive function in the absence of inflammatory factors and TGF-β (Fontenot et al., 2017). The development of Th17/Treg balance towards Th17 is considered an important feature of the inflammatory response as well. More importantly, the extended Th1/Th2/Th17/Treg cell pattern could be essential to understand inflammatory mechanisms at the molecular level.

## 3 Exosomes

### 3.1 Structure and components of the exosomes

Exosomes are membrane-bound vesicles with a similar structure to the plasma membrane, containing miRNA, mRNA, and proteins and being capable of transferring from the original cells to the corresponding target cells *via* body fluids (Jia et al., 2017). It has been confirmed that the nasal lavage fluid contained exosomes and the components have been identified to be surface markers CD9, CD63 and CD81 using flow cytometry (Lässer et al., 2011). These findings are to compare the difference in exosome components in nasal lavage fluid between normal persons and CRSwNP patients.

### 3.2 Generation and exportation of exosome

Exosomes are microvesicles with diameter in 30–100 nm and morphologically with a “dish” morphology consisting of a lipid bilayer. Their precursors are luminal vesicles (ILVs) in cellular polyvesicles (MVBs), and after MVB fusion with the cell membrane the precursors are released into the extracellular form of exosomes.

**FIGURE 2:**
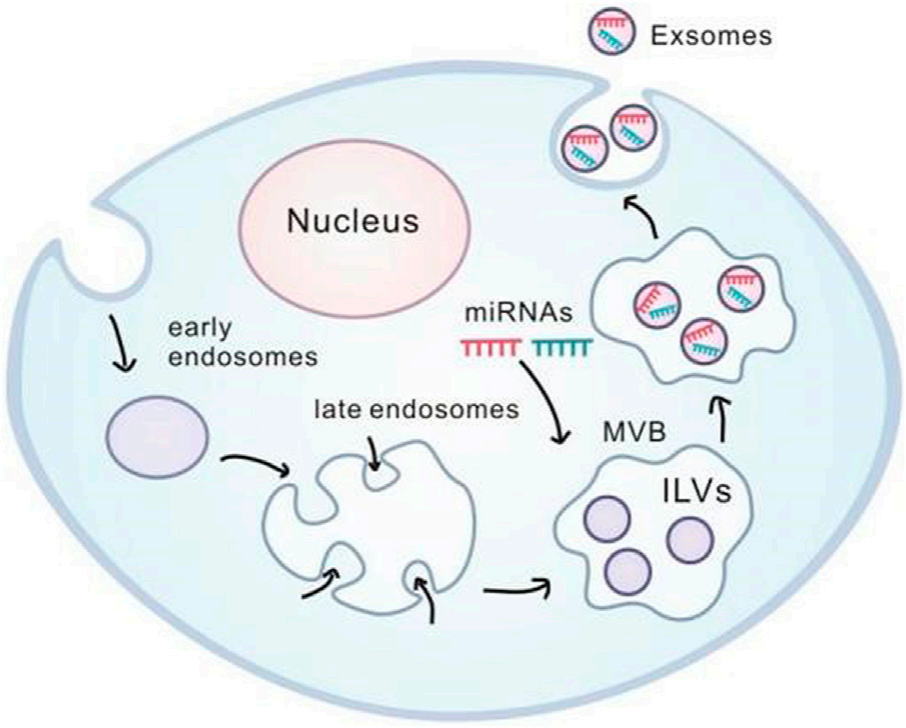
Generation and internalization of exosome.

Exosomes contain receptors and transmembrane proteins on the cell membrane, and their formation starts as early endosomes formed by cell membrane depressions. Following that, they gradually become a late endosome, budding into multiple vesicles (MVBs), and selectively transporting intracellular proteins and specific miRNA to the luminal vesicles (ILVs). Subsequently, the multi-vesicle bodies fuse with the cell membrane to release luminal vesicles outside the cell and thereby become exosomes. Exosomes secreted by cells can be transported through extracellular fluid such as serum, lymph and taken up by target cells, accordingly causing biological consequences (Jia et al., 2017). However, the involvement of exosomes in signaling between inflammatory cells, as well as the migration of nasal polyps and inflammatory responses has been less explored. It is plausible to speculate that the understanding exosomes-mediated intercellular signaling may provide essential information for pathogenesis and clinical therapy for the nasal polyps.(Figure 2)

### 3.3 Differential expression of miRNA in CRSwNP

Significant difference in exosomes miRNA levels were detected between normal persons and CRSwNP patients. In the study of Xuan and Xia, the levels of miR-155, miR-375, miR-671-3p, and miR-92a-3p were associated with CRSwNP development *via* the same controls (Xia et al., 2015; Xuan et al., 2019). Analysis on GO enrichment and KEGG pathways showed that 192 of miRNA expression was significantly downregulated, but no substantial upregulation was observed in CRSwNP relative to normal control (Yu et al., 2021). Therefore, it could be concluded that the occurrence of CRSwNP is associated with the downregulation of miRNAs, suggesting that miRNAs with significant alteration in the expression levels could contribute to physiolpathology and be potentially developed as therapeutic targets for clinical therapy of CRSwNP patients.

## 4 Alteration of miRNAs levels in the inflammatory cells in response to inflammation stimuli

CRSwNP mainly showed an inflammatory response in Th2 cells, along with the phenomenon of eosinophil infiltration. The level of the inhibitory cytokine IL-10 was able to affect CRSwNP progression, and the TNF-α expression is also important for CRSwNP progression. Some miRNA were found to influence the occurrence and progression of nasal polyps by regulating the levels of IL-10 or TNF-α.

### 4.1 The miRNA-21 was able to increase the IL-10 levels in the nasal epithelial cells to inhibit the inflammatory response

The miR-21 functions as negative feedback regulator for the CRSwNP *via* reducing the levels of proinflammatory cytokines. By targeting to PDCD4 mRNA, the miR-21 inhibited PDCD4 translation, thereby reducing the P65 phosphorylation of NF-kB and consequently repressing the inhibitory effect of nuclear IL-10 gene expression and elevating IL-10 levels. IL-10 could inhibit the expression of proinflammatory cytokines such as IL-25, IL-33, TSLP, IL-1, IL-6 and IL-8, thus inhibiting the progression of inflammatory response (Liu et al., 2021a). Therefore, the miR-21 confers anti-inflammatory effect *via* directly elevating the level of IL-10 targeting to the miR-21/PDCD4/NF-kB pathway thereby downregulating the expression of inflammatory factors.

### 4.2 Elevation of miR-142-3p level enhances TNF-α expression in nasal epithelial cells and exaggerates the inflammatory response

Co-elevation of both miR-142-3p and TNF-α levels were observed in human nasal epithelial cells upon stimulation with LPS in a way that the abundance of both miR-142-3p and TNF-α were positively correlated. It has been acknowledged that co-upregulation of both miR-142-3p and TNF-α in nasal epithelial cells could enhance the inflammatory response. The related molecular mechanism is the induced expression of TNF-α by the elevation of miR-142-3p promotes the release of IL-6, IL-10, and IFN-γ from monocyte macrophages and eosinophils, whereas induced release of NF-kB, C-fos and C-Jun subsequently regulates the inflammatory response, and *vice versa* (Yang et al., 2018). Therefore, it could be speculated that miR-142-3p might be involved in the inflammatory response through the LPS-TLR-TNF-α signaling pathway (Qing et al., 2021).

### 4.3 The miRNA-19a down-regulates IL-10 levels in peripheral dendritic cells and promoted the inflammatory response

In response to TLSP, peripheral dendritic cells (DCs) activate and present antigen and costimulatory signals to differentiate naive T cells into effector Th2 cells, while secrete IL-10 to reduce the inflammatory response. Significantly upregulated level of the IL-4 due to elevated miR-19a levels in CRSwNP patients could suppress IL-10 expression in dendritic cells. Furthermore, IL-4 suppresses the transcription of the IL-10 gene in response to HDAC11, the gene transcriptional repressor (Luo et al., 2017), thus significantly increasing the levels of miR-19a and IL-10 in DC cells of CRSwNP patients (Simpson et al., 2014).

## 5 Alteration of miRNAs in CRSwNP effects airway remodeling

### 5.1 Airway remodeling in the CRSwNP

In CRSwNP, airway remodeling is mainly characterized by fibrin accumulation, increasing mucin-containing mucus and epithelial mesenchymal transition. The increase of coagulation factor expression and decreased fibrinolytic activity in nasal tissue induced the accumulation of fibrin in nasal polyps. Th2 cytokines in CRSwNP can inhibit t-PA expression in nasal epithelial cells and inhibit fibrin degradation, resulting in edema eosinophilic nasal polyps in type 2 inflammation, and the increase of coagulation factor XIIIa in type 2 nasal polyps contribute to fibrin accumulation as well (Takabayashi et al., 2013a; Takabayashi et al., 2013b). Type 2 inflammatory cytokines such as IL-4, IL-13, IL-8, and IL-33 in CRSwNP patients enhanced expression of MUC5AC in nasal polyp-derived epithelial cells (Zhang et al., 2019). In response to IL-13, the expression of anion transporter pendrin protein was increased in the epithelial cells of CRSwNP, leading to the increased mucus production (Seshadri et al., 2015). EMT causes loss of cell connectivity and polarity in CRSwNP tissues, conversion of nasal epithelial cells to mesenchymal cell phenotypes, downregulation of epithelial markers expression such as e-cadherin and SIRT1 and upregulation of mesenchymal markers expression such as-SMA and MMP (Thiery et al., 2009). Of the markers, MMP can degrade specific extracellular matrix (ECM) components. Indeed, significant elevation of MMP2 and MMP9 levels observed, while the TIMP1 and TIMP4 levels are significantly attenuated in CRSwNPs, which is closely related to the occurrence of polyps in CRSwNP (Li et al., 2010).

### 5.2 Airway remodeling-related miRNA in CRSwNP

#### 5.2.1 Upregulation of miR1555p promotes EMT

Elevation of miR155-5p levels in nasal epithelial cells caused by enhanced TGF-β1 levels in CRSwNP patients could more efficiently target to SIRT1, participate in EMT progression in epithelial cells and affect the airway remodeling process in nasal tissue (Yang et al., 2020). This is further evidenced by the factor that a miR155-5p inhibitor can attenuate the effect on SIRT1 and subsequently inhibit EMT, thus surpressing the polyposogenesis of CRSwNP.

#### 5.2.2 TGF-β1 induces the nasal epithelial cell-mesenchymal transformation *via* miR-21

TGF-β1, a representative cytokine in tissue remodeling, could activate EMT signaling to initiate tissue remodeling of the airway epithelium and nasal tissue, leading to the altered epithelial cell morphology and upregulation of mesenchymal protein and miR-21 expression. Previous studies have observed significant upregulation of both TGF-β1mRNA and miR-21 in CRSwNP patients relative to normal control group. Consistently, the miR-21 inhibitor and an Akt-specific inhibitor could inhibit TGF-β1-induced EMT, whereas the reduction in miR-21 abundance was accompanied by elevation of the PTEN levels and decrease in Akt phosphorylation (Li et al., 2019). Therefore, it is reasonable to suggest that miR-21 or the TGF-β1-miR-21-PTEN-Akt axis may potentially developed as targets for clinical therapy of CRSwNP.

#### 5.2.3 miR29b3p promotes α-tubulin deacetylation-mediated tissue remodeling

MMP9 is able to bind to integrin proteins and regulate α-tubulin acetylation and deacetylation. Acetyl-α-tubulin could affect cell morphology and got involved in the formation of nasal polyps in CRSwNP. MiR29b-3p and TMP1 play counterpart roles in terms of MM9 expression regulation in ways that miR-29b-3p stimulates expression of MMP9, while TIMP1 reduces MMP9 abundance. miR29b3p affects acetyl-α-tubulin levels by enhancing the interaction between MMP9 and integrin 1, the lack of which is associated with epithelial mesenchymal transformation induced by TGF-β (Liu et al., 2021b). Thus, regulation of α-tubulin acetylation levels by miR-293p could be another promising strategy for the treatment of nasal polyps (Table 2).

**TABLE 2:** Effect of some miRNA on the immune response and airway remodeling.

Effect of the inflammatory cell miRNA levels	
Inflammatory response	The miR-21/PDCD4/NF-kB pathway regulates IL-10 together with pro-inflammatory factor levels
	miR-142-3p enhances TNF-α levels through the LPS-TLR-TNF-α signaling pathway to promotes the inflammatory response
	The miRNA-19a decreased IL-10 levels in peripheral dendritic cell promoted the inflammatory response
Airway remodeling	Increasing miR1555p levels promote EMT
	Regulation of miR-21 levels or affecting a portion of the TGF-β1-miR-21 -PTEN-Akt axis is important for the treatment of CRSwNP
	miR29b3p promotes a-tubulin deacetylation-mediated tissue remodeling

## 6 The miRNA is secreted to the exosome machinery

### 6.1 The role of RNA-binding protein in miRNA secretion

Cells selectively transport miRNA into exosomes, and the proteins involved in this process mainly include RNA-binding proteins such as hnRNPA2B1 with Argonaute-2, as well as exosome assembly-synthesized membrane proteins such as caveolin-1 and sphingophospholipase 2. RNA-binding protein (RBP) is a class of proteins that can specifically bind RNA molecules to mediate their entry into exosomes. Studies have found significant differences in concentrations between the intracellular miRNA and the secreted miRNA, indicating the essential role of RBP in miRNA entry into exosome.

### 6.2 The RNA-binding protein involved in the regulation of miRNA secretion to the exosomes

A number of RBPs have been characterized to be associated with the secretion of miRNA into exosomes, including hnRNPs, Ago2, YBX-1, MEX3C, MVP, and La proteins. hnRNPs play diversified roles in regulating transcriptional and post-transcriptional gene expression, Including RNA splicing, polyadenylation, capping, modification, export, localization and translation. hnRNPs bind to short RNA single strands through specific domains. For example, hnRNP-q contains a N-terminal domain that recognizes the GGCU sequence highly affinitive to the miRNAs. The miRNA binding domain in hnRNP-q could initiate and regulate transportation of miR-3470a and miR-194-2-3p into exosomes (Santangelo et al., 2016). In consideration of the essential roles of hnRNP-q, hnRNP-q downregulation could repress miRNA transportation into exosomes, leading to accumulation of miRNA in the cytoplasm.

Like hnRNP-q, ARGONAUNT2 (AGO2) is highly affinitive to miRNAs and can serve as carrier for transportation of miRNAs into exosomes. The AGO2 mediated miRNAs transportation is regulated by the KRAS-MEK-ERK pathway *via* alteration of the AGO2 phosphorylation levels. This was confirmed by the elevated miR-100 content of hyperphosphorylated KRAS mutations *in vitro* (Cha et al., 2015). The RNA-binding domain of YBX-1 enables it to interact with miR-133 and miR-223 for transportation of both miRNAs into exosomes. To provide evidence, reducing YBX-1 expression levels in H/R EPC reduced the abundance of miR-133 in exosomes, while the miR-133 abundance in the cytoplasm remains largely unaltered (Lin et al., 2019). Unlike hnRNP-q and AGO2 with miRNA binding domains for miRNA transportation into exosomes, it was found that MEX3C is not a direct target of miR-451a during miR-451a transportation to exosomes. The RBP MEX3C was involved in forming the MEX3C-Ago2 complex, mediating miR-451a entry into exosomes (Lu et al., 2017). This finding suggests the two RBP involved participation in the process of miRNA transport into exosomes, providing new perspectives on miRNA transport. Another RBP, Dome principal protein (MVP) is involved in the transport of RNA from nucleus to cytoplasm and from cytoplasm to exosomes. MVP overexpression in CT26 colon cancer cells leads to decreased abundance of miR-193a in the cells and increased levels in exosomes, suggesting the important role the MVP plays in the miR-193a transportation to exosomes. The La protein, a transcription factor of the RNA polymerase III and a RBP, was found to play important role in transportation of some miR-122 into exosomes. Downregulation of the La content in the cytoplasm would cause a decrease in miR-122 abundance in exosomes and *vice versa* (Temoche-Diaz et al., 2019).

### 6.3 The HNRNP-A1 recognizes the miRNA containing the AGAGGG motif and allows for delivery to the exosomes

**FIGURE 3:**
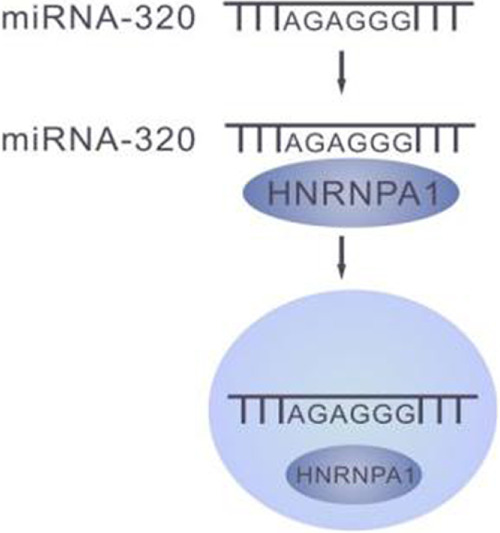
HNRNPA1 mediates transportation of the miRNAs with AGAGGG motif into the exosomes.

HNRNP-A1 was found to specifically recognize miRNAs that contain AGAGGG motifs such as miR-320 to mediate miRNA secretion to exosomes. Upon knockdown of HNRNPA1, miR-320 level increased significantly in the cytosol, and accordingly dramatic decrease in exosomes, suggesting the essential role of HNRNPA1 in maintenance of some miRNAs accumulation in exosomes (Gao et al., 2019). This further suggests that the synchronously dynamic fluctuation of miRNAs in cells and exosomes is mediated by RNPs. (Figure 3)

### 6.4 Membrane protein caveolin-1 is involved in HNRNPA2B1 regulation of miRNA transport to exosomes

**FIGURE 4:**
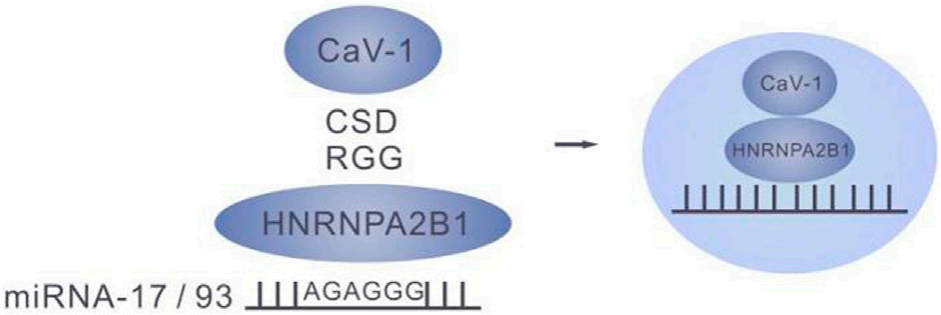
The cav-1/HNRNPA2B1 complex.

RBPs, such as hnRNPs, Ago2, YBX-1, YBX-1, MEX3C, MVP and La proteins all bind miRNA and facilitate their transfer to exosomes. The membrane protein caveolin-1 was involved in the RBP- mediated miRNA entry into exosomes. Upon induction of oxidative stress, Y-14 of caveolin-1was phosphorylated in the lung epithelial cells to enable the CSD of caveolin-1 to interact with the RGG of HNRNPA2B1 to form a cav-1/HNRNPA2B1 complex (Lee et al., 2019). Importantly, previous study demonstrated the involvement of membrane proteins in the RBP-mediated entry of miRNA into exosomes.(Figure 4)

## 7 Effect of exosome-delivered miRNA on target cells

### 7.1 The miR-22-3p in the CRSwNP exosomes regulates vascular permeability by targeting the VE-calcium adhesion proteins

MiR-22-3p targets mRNA 3′-UTR, coding for VE-calcium adhesion protein. Significant upregulation of miR-22-3p expression in nasal lavage fluid of CRSwNP patients could downregulate expression of VE-cadherin, leading to abnormal vascular permeability and consequently causing the aberrant tissue edema and nasal polyp growth (Zhang et al., 2020). Different study further supports the conclusion that miR-22-3p in exosomes downregulates expression of the VE-calcium adhesion proteins to regulate CRSwNP occurrence and progression (Gu et al., 2017). Altogether suggests that exosome-delivered miRNA is capable of regulating CRSwNP progression *via* targeting adhesion protein molecules。

### 7.2 Exosome-delivered miR-17/93 is able to downregulate Irf2bp2 in macrophages to activate them

**FIGURE 5:**
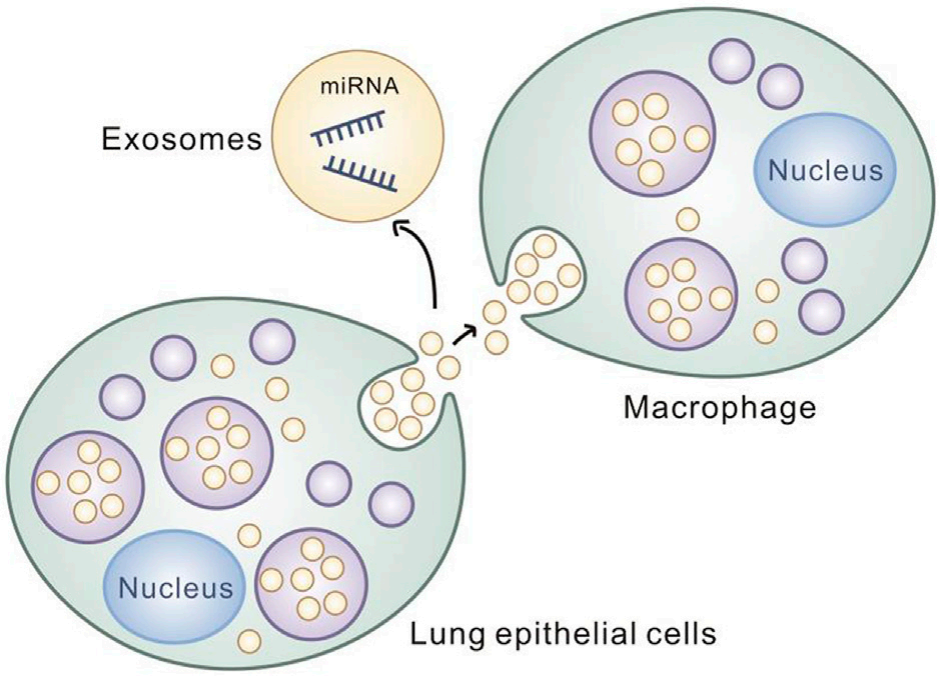
Lung epithelial cells transmit inflammatory signals to macrophages through exosomes.

It was found that miR17/93-containing exosomes released by lung epithelial cells under oxidative stress were able to be ingested by macrophages, and thedelivered miR17/93 was able to reduce Irf2bp2 levels in macrophages. The Irf2bp2 suppresses macrophage activation, and the reduced level of Irf2bp2 in macrophages can activate macrophages and promote macrophage migration and the synthesis and release of cytokines such as TNF and IL-1 (Chen et al., 2015). It is confirmed that exogenously delivered miRNA can regulate the expression level of intracellular related proteins followed by causing the corresponding biological consequences. It is of great significance for miRNA transmission in CRSwNP to explore the occurrence mechanism and to develop clinical therapy for CRSwNP. (Figure 5)

## 8 Future

CRSwNP is an inflammatory respiratory disease with high global incidence and high recurrence, and limited information for its pathogenesis is available. Nasal epithelial cells, myeloid dendritic cells, ICL2 cells, eosinophils, mast cells, macrophages and Th2 cells are all involved in the inflammatory process of CRSwNP. The new Th1/Th2/Th17/Treg cell pattern opened a novel angle towards the mechanism of inflammatory response and progression.

Changes in cellular endogenous miRNA content have important effects on both inflammatory cytokine production and airway remodeling in CRSwNP. Regulation of intracellular miRNA levels has significant potential in treating CRSwNP and studying the pathogenesis of CRSwNP.

Given that the exosomes could be isolated from the Nasal lavage fluid in CRSwNP patients and as an easy way miRNAs could be profiled to identify and characterize, the exosomes and the contained miRNAs have become an efficient tool convenient for investigating the miRNA-mediated pathology for CRSwNP.

Exosome-delivered miR-22-3p in CRSwNP regulates vascular permeability by targeting ve-calcium adhesion proteins, and the exosome-delivered miR-17/93 in lung epithelial cells is able to downregulate Irf2bp2 in macrophages to activate macrophages. Cell-secreted exosomes have been shown to regulate the inflammatory response in the target cells, of which miRNA plays an important role. Extracellular stimulation signals transmitted by miRNA in exosomes can activate or inhibit inflammatory cells, cause intracellular-related biological events such as generation and release of the related inflammatory cytokines, and regulate the occurrence and progression of CRS. The study of miRNA in exosomes provides new angle for the development of novel drugs for therapy of CRSwNP.

Studies on the process of RBP in miRNA transport to exosomes revealed the causes of synchronized changes between endogenous miRNA and foreign miRNA in exosomes. Moreover, RBP is important for the entry of endogenous miRNA into exosomes. Several specific membrane proteins such as caveolin-1 have also been shown to be involved in RBP-mediated miRNA entry into exosomes, miRNA entry processes may involve more intracellular components, and miRNA transport into exosomes with greater research potential.

**FIGURE 6:**
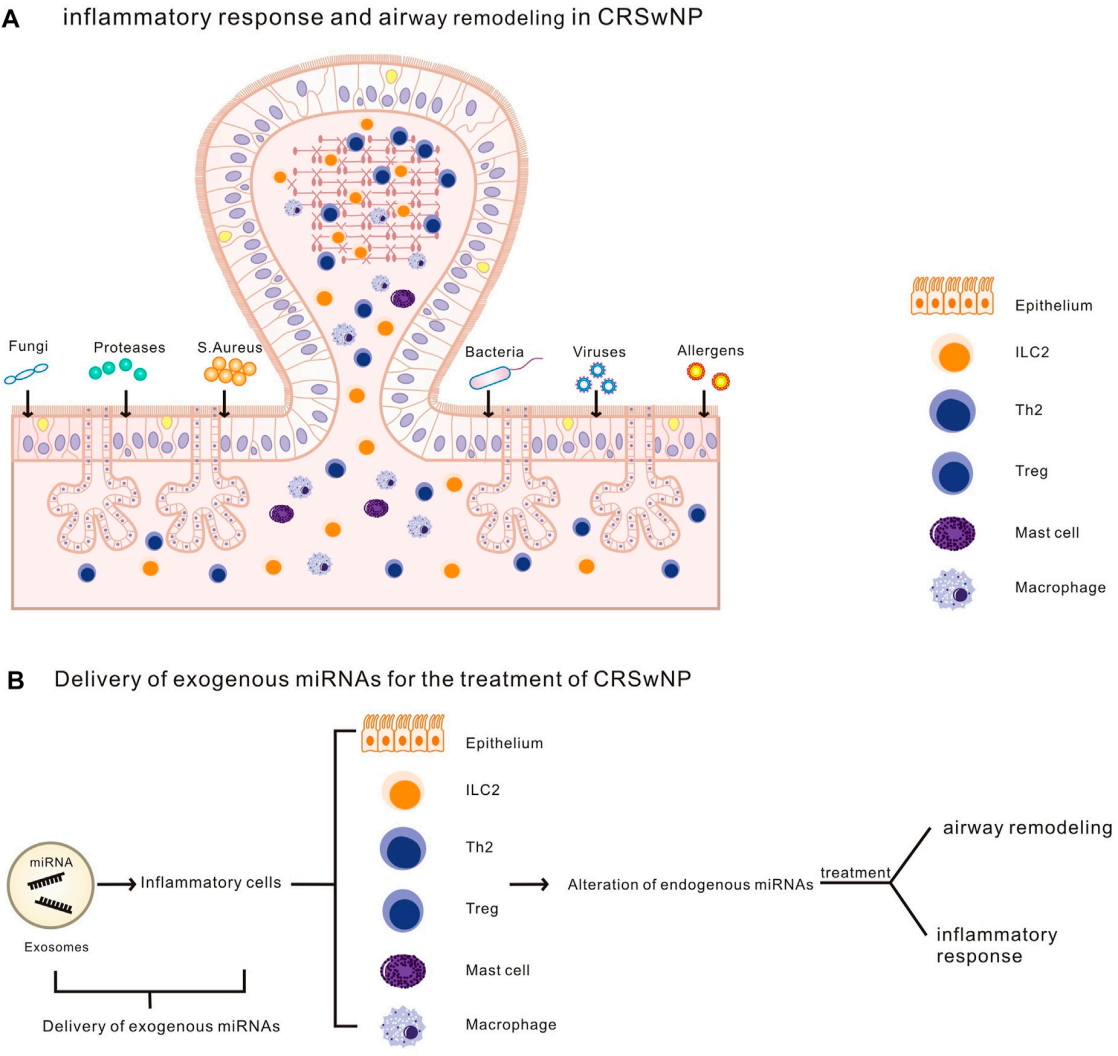
**(A)**. Airway remodeling and an inflammatory response in CRSwNP.**(B)**.Exosomes deliver exogenous miRNA to inflammatory cells (including nasal epithelial cells and other CRSwNP-related inflammatory cells), altering cellular endogenous miRNA levels, regulating airway remodeling and inflammatory response in CRSwNP.

Altered miRNAs levels have important effects on inflammatory progression and airway remodeling in nasal polyps. Exogenous exosome-delivered miRNAs targets nasal mucosal epithelial cells as well as associated inflammatory cells in CRSwNP, changes their cellular endogenous miRNA content, and regulates the occurrence and progression of CRSwNP by regulating inflammatory responses and airway remodeling. This provides new ideas and methods to study the pathogenesis, treatment and prevention of nasal polyps, as well as to realize the precise treatment of nasal polyps and reduce the recurrence rate of nasal polyps after surgery (Figure 6).
